# Significant variation in salivation by short-term suggestive intervention: a randomized controlled cross-over clinical study

**DOI:** 10.1186/1746-160X-10-49

**Published:** 2014-11-27

**Authors:** Maximilian Satzl, Albrecht Schmierer, Florian Zeman, Gottfried Schmalz, Thomas Loew

**Affiliations:** Department of Psychosomatic Medicine, Department of Operative and Restorative Dentistry, Periodontology, and Pedodontics, University Medical Center Regensburg, Franz-Josef-Strauss-Allee 11, 93053 Regensburg, Germany; Chairman of the German Society of Dental Hypnosis, Stuttgart, Germany; Center of Clinical Studies, University Medical Centre of Regensburg, Franz-Josef-Strauss-Allee 11, 93053 Regensburg, Germany; Department of Operative Dentistry and Periodontology, University Medical Centre of Regensburg, Franz-Josef-Strauss-Allee 11, 93053 Regensburg, Germany; Department of Psychosomatic Medicine, University Medical Centre of Regensburg, Franz-Josef-Strauss-Allee 11, 93053 Regensburg, Germany

**Keywords:** Salivary flow, Complimentary medicine, Randomized controlled clinical trials

## Abstract

**Introduction:**

Most dental procedures require a dry working environment. Although many evaporative drying methods are available, an additional reduction of salivary flow would often be helpful.

**Methods:**

This prospective randomized cross-over study compares salivary production in 31 volunteers during direct, indirect, and non-suggestive (control group) intervention. Overall, each volunteer underwent four salivation measurements, i.e. two measurements during two different types of hypnotic suggestion (indirect and direct) arranged in random order and two control sections. All four measurements were conducted successively.

**Results:**

Both suggestive methods significantly reduced salivary production in comparison to the two control sections (direct suggestion Δ = 1.46 grams per 5 min, p < 0.001, indirect suggestion Δ = 0.94 grams per 5 min, p = 0.039). Direct suggestion showed a significantly higher reduction of salivary production than indirect suggestion (Δ = -0.53 grams per 5 min, p = 0.001).

**Conclusions:**

Hypnotic suggestion represents a simple and inexpensive method to reduce salivation and could thus create a better working environment for more comfortable dental treatments for both patients and dentists.

## Introduction

Salivation is one of the most important requirements for oral health [1, 2]. Produced by the large salivary glands (glandula sublingualis, glandula submandibularis, and glandula parotis) and the minor salivary glands of the palate, tongue, lips, and cheeks, saliva consists of 99% water and 1% organic and inorganic molecules [3, 4]. Saliva is sterile before leaving the glands, but immediately mixes with crevicular fluid, food remains, microorganisms, etc. when entering the oral cavity [5]. The composition of saliva not only facilitates ingestion and deglutition but also prevents oral diseases associated with xerostomia [6–8]. Controlled by the autonomous nervous system, saliva production ranges between 500 ml and 700 ml per day and alternates between 0.25 and 0.35 ml/min during sleep. After electrical or mechanical stimulation, saliva production may rise up to 1.5 ml/min. The highest amount of saliva is produced around meal times, peaking at noon, and the lowest amount during sleep [9]. In the case of dental treatments, however, increased salivation complicates most interventions because of the presence of moisture and bacteria [10]. Therefore, dentists try to establish dry oral conditions to achieve better results in dental restorations, dental impression, root canal therapy, or diagnosing. Conventional techniques of removing saliva are absorbent cotton or suction, and the rubber-dam method that keeps saliva away from the working area [11–13]. However, these methods only hold off or remove existing saliva. Reducing salivation at its source by means of suggestion could simplify dental treatment, either by replacing the above-mentioned methods or by at least supporting them.

Hypnosis represents an altered psycho-physiological state of consciousness, increasing absorption and focused attention, decreasing peripheral awareness, and reducing spontaneous thinking [14–16]. The induction procedure consisting of a set of verbal instructions leads to this specific mental state that includes a change in baseline mental activity [17]. The intensity of the hypnotic state varies among individuals. For example, the hypnotic responsivity of children younger than 8 years of age is significantly higher than that of older children or adults [18]. The use of specific suggestions leads to the typical ‘hypnotic’ phenomena, such as alterations in sensory experience and motor control, amnesia, and a falsified perception of oneself and the environment [19]. Suggestibility, the willingness to accept suggestions, will thus be increased [17].

Hypnosis has been shown to modify visual perception [20], auditory perception [21], attention [22], intention [23], and awareness of control [24]. Moreover, hypnosis is a simple and effective method to control pain, fear, and anxiety [15, 25]. Controlling such emotions is the most common indication for using hypnosis in dental practice because hypnotic interventions have been proven to be effective in reducing anxiety and pain in invasive medical procedures [26]. Additionally, several studies have proven the influence of hypnosis on heart rate and blood pressure and on inducing local anesthesia and hemorrhage [27].

Generally, hypnotic suggestion in dentistry has been used for treating children or patients afraid of dental therapy [28]. This study aims at modifying physiological parameters for reducing the salivation rate for more comfortable treatments in conjunction with the calming effects of hypnotic suggestion [27]. The objective of this study is to determine the extent to which salivary production may be reduced by standardized suggestive interventions. Thus, we compared two groups of volunteers who received the two different types of suggestion available with a control group who did not receive any suggestive method (baseline).

## Materials and methods

### Volunteers

31 volunteers, 18 women and 13 men, mainly fellow students, friends, and relatives of the author (MS), were recruited between January 2011 and April 2011. Participants had to be at least 16 years old and should not have any significant medical problem, such as reduced salivary flow due to disease or radiation therapy. Because of the cross-over design of our study and the comparison of the measured data, we did not specify any further exclusion criteria with regard to a possible reduction of salivation. No upper age limit was set. All 31 volunteers fulfilled these criteria and participated in the study. Before starting the measurements, all participants had to sign an informed consent form and had to fill in a questionnaire on medication, smoking, chronic disease, salivary problems, and previous radiation therapy. Prerequisites for a suitable measuring environment were the absence of any distraction for the human senses as well as a comfortable room temperature.

### Suggestive method

We applied two different methods of suggestion (direct and indirect) including background trance music [29]. The indirect method used metaphors, such as describing hot and dry environments or situations, whereas the direct suggestion method included various instructions to stop salivation. The texts, lasting 15 min each, were elaborated and spoken by one of the authors (AS), a dentist and expert hypnotherapist practicing in Stuttgart, Germany. The recordings ─ made available to the volunteers by ear-phones ─ were of equal length for reasons of repeatability. Both texts, translated into English by a native speaker, can be found in the Appendix. After each suggestion, a 2-min text of de-suggestion was played to get the participants released from their state of trance.

### Salivary collection method

According to an experiment by Navazesh et al., we used the draining and spitting method because of its high reproducibility and its low standard error of the mean [30]. For this method, the volunteers were instructed to tilt their heads forward, keep their eyes open, and avoid movements ─ including swallowing ─ as far as possible. Simultaneous dental treatment was not possible during this collection method but we considered accurate measurement more important. First, the test persons had to swallow any saliva present at the start of the recording phase. Any saliva produced within 5 min after swallowing would be collected by letting the saliva drain into a cup held close to the open lips. In the final stages of a 5-min collection procedure, the volunteers were asked to spit the remaining saliva into a measuring jug.

The quantitative determination included weighing the measuring cup minus its basic weight and inscribing the name of the test person. Each volunteer used one cup for each of the four measurement rounds. Saliva obtained during the preceding measurements remained, was also numbered among basic weight, and accordingly subtracted before the next measurement round. Because of the effect of evaporation, the weight of saliva decreased during the testing procedure. For this reason, baseline measurements were taken directly before starting a new measurement round. Navazesh et al. showed in pilot studies that weight determination was more reliable than volume determination [30].

### Testing procedure

**Figure 1:**
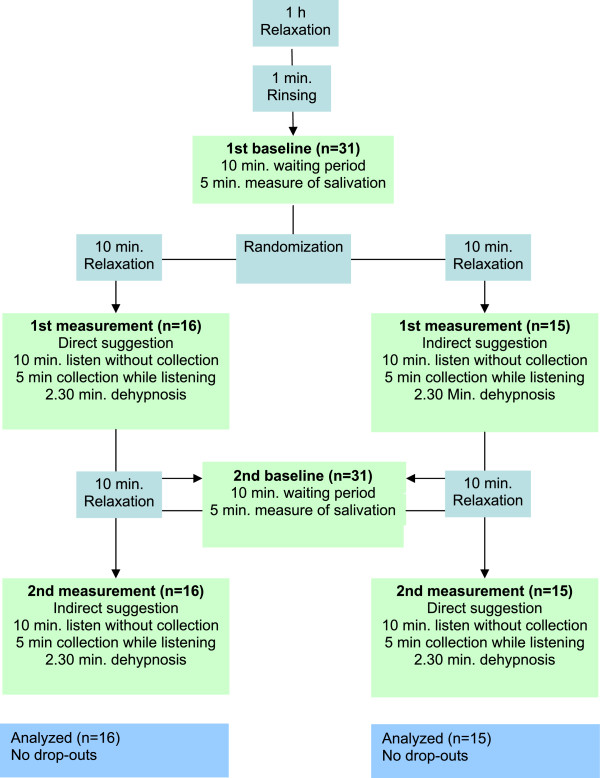
**Patient flow**-**diagram.**

The testing procedure took approximately 1 h as well as 1 h of relaxation beforehand. For best possible supervision, the measurements took place in groups of no more than five volunteers. Each volunteer underwent the four recording phases within 1 h. The participants should not have eaten anything for at least 12 h on the day of testing and also had to refrain from smoking. The procedures of each recording phase were the same. When presenting at the location, the volunteers were offered 1 h to relax and to familiarize themselves with the procedure and the surroundings. Afterwards, the volunteers were asked to rinse their mouth with de-ionized water for 1 min to remove food debris and other non-salivary elements, such as crevicular fluids, oral microorganisms, and host cells [31] that may stimulate salivary flow and thus falsify the measurements.All 31 volunteers underwent all four types of recording (double baseline, direct (A) and indirect (B) suggestion). Baseline data were recorded twice, i.e. in the first and third recording phase, and the suggestive methods were measured once in the second phase and once in the fourth phase. Each testing method was followed by a wash-out phase of 10 min. After the first baseline measurement of 5 min, the volunteer was randomized into one of the two testing procedure groups (A-B vs. B-A) according to a predefined randomization list. After listening to a suggestion for 10 min, the volunteer immediately underwent a 5-min saliva collecting phase while the audio tape continued to play. A detailed participant flow-diagram is shown in Figure 1.

After the measurements, the volunteers were asked about their well-being during the suggestive intervention. Answers to this question included “better”, “inferior than before”, or “unaltered”. The volunteers were also asked about their favorite suggestive method (direct or indirect) and if they would like to listen to the same or a similar suggestion during dental treatment and why (yes or no and, if possible, why). When asked again about their well-being over the past few months, all volunteers stated that they had not experienced any physical or psychological problems.

### Ethical considerations

The Ethics Committee of the University Medical Center Regensburg, Regensburg, Germany, approved of the experimental design, and the test-persons gave their consent in written form. The study was conducted without any conflicting interests and was not supported financially.

### Statistical analysis

Baseline characteristics were summarized by suggestion sequences (direct and indirect vs. indirect and direct). Continuous variables are presented as mean values and standard deviations; categorical variables are presented as absolute numbers and proportions. The effect of the type of suggestion on salivation was assessed using a linear mixed model. A linear mixed model is a generalization of the traditional repeated measures ANOVA. It has the advantage to show both fixed and random effects, to deal with missing values, and to provide estimated marginal means adjusted for all additional variables within the model. Volunteers within a recording phase were included into the model as a random effect. To analyze carry-over and period effects, phases and periods were included into the model as fixed effects. Because of the short washout phase, only the first baseline measurement was used as a reference. Nevertheless, the model was adjusted for the difference between the two baseline measurements by adding as an additional covariate [32]. For all analyses, a p-value <0.05 was considered statistically significant. All statistical analyses were done with the SAS version 9.3 (SAS Institute, Inc., Cary, NC) using PROC MIXED for the linear mixed model.

## Results

**Figure 2:**
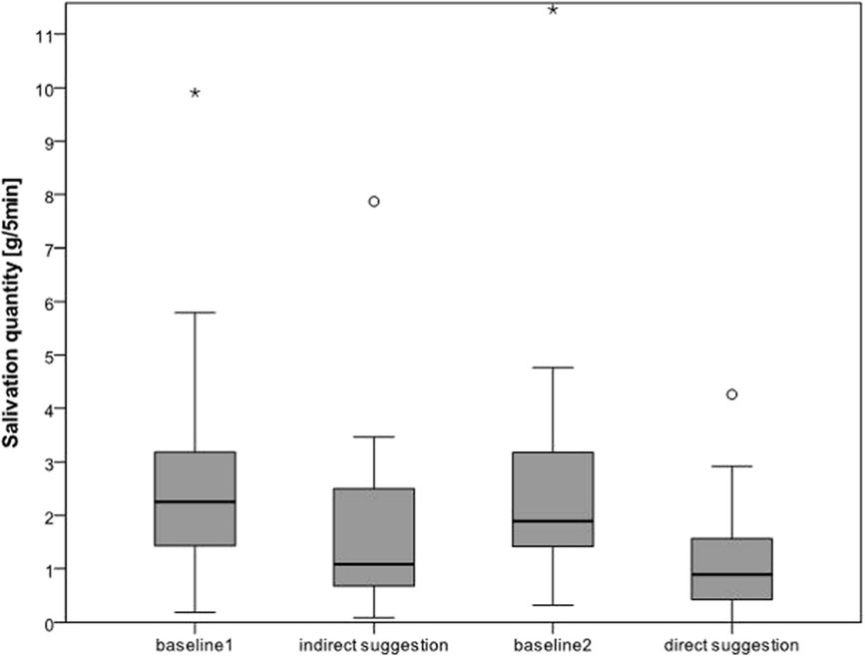
**Boxplots of salivation quantity for baseline and suggestion measurements.**

**Figure 3:**
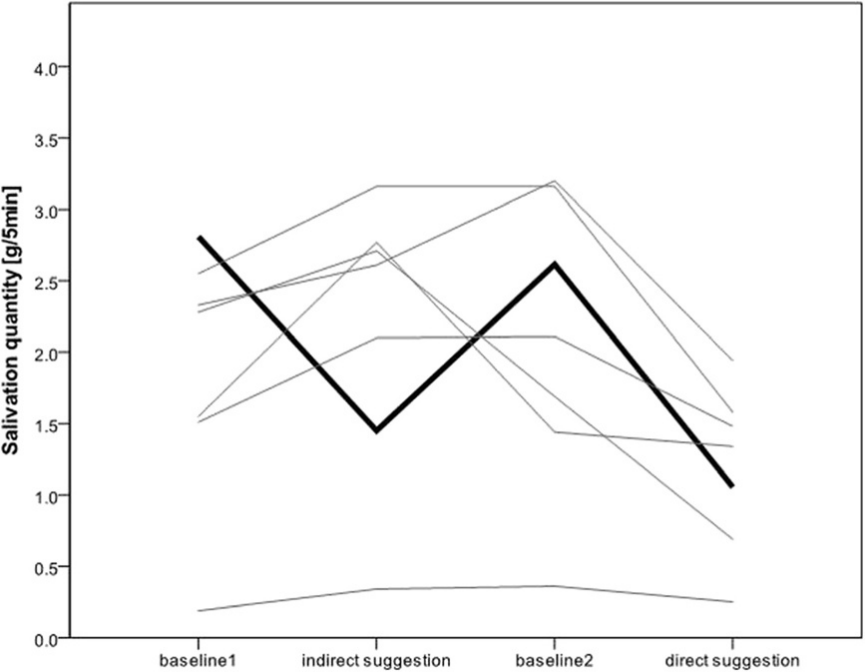
**Salivation quantity on subject basis for baseline and suggestion measurements.** The bold black line represents the mean salivation rate of all subjects reacting on both suggestion methods. The grey lines represent individual subjects not reacting on one or both suggestions.

Volunteers were aged between 18 and 63 years. Table 1 presents the demographic data of the patients. Compared to the first and second baseline measurements, both direct and indirect suggestion significantly decreased salivation. Direct suggestion showed a significantly higher decrease in salivation than indirect suggestion (Figure 2, Table 2). In a subsequent interview, all volunteers (100%; 31/31) were asked about their well-being during the suggestion, if they had felt comfortable, and if they would like to listen to such texts during dental treatment. None of the volunteers had felt disturbed or anxious. When asked about their favorite type of suggestion, 80% (25/31) preferred indirect suggestion. As a reason for their preference, 72% of them (18/25) stated that they had become part of the narrative, whereas the remaining volunteers (28%, 7/25) did not have any explanation for their decision.22 volunteers showed the expected pattern of reduced salivation (baseline 1 high, suggestion low, baseline 2 high, suggestion low). 7 volunteers only showed lower salivation values in one type of suggestion (5 volunteers at direct suggestion, 2 at indirect suggestion), whereas 2 volunteers had an increased amount of salivation in both suggestions compared to baseline (Figure 3). In three volunteers, salivary production could even be completely stopped during the 5 min recording phase during the direct suggestion method.

**Table 1:** **Baseline characteristics**

	All, 31 patients	Sequence direct- indirect, 16 patients	Sequence indirect- direct, 15 patients
**Age** (years), mean (SD)	34 (12)	36 (15)	32 (10)
**Baseline salivation**, mean (SD)	2.6 (1.9)	2.5 (1.3)	2.6 (2.4)
**Sex** (N, %)			
Male	13 (42%)	6 (37.5%)	7 (47%)
Female	18 (58%)	10 (62.5%)	8 (53%)
**Smoker** (N, %)	5 (16%)	3 (19%)	2 (13%)
**Medication** (N, %)	1 (3%)	0 (0%)	1 (6%)
**Diseases** (N, %)	1 (3%)	1 (6%)	0 (0%)

**Table 2:** **Linear mixed model**

	Baseline, first period	Direct suggestion	Indirect suggestion
Salivation [ml]	2.55 (SD 1.88)	1.09 (SD 0.96)	1.62 (SD 1.53)
ΔSalivation [ml]		1.46 (SD 2.08)	0.94 (SD 2.42)
p-value		<0.001	0.039
ΔSalivation between direct and indirect suggestion, [ml]		-0.53 (95%-CI: -0.83, -0.23)*	
p-value		0.0012	

Period effect: p = 0.28, carryover effect: p = 0.38, *Estimated according to the linear mixed model, 95%-*CI*: 95%-Confidence interval.

## Discussion

The literature includes many studies on the correlation between salivary flow and suggestive intervention but with different results. Some studies showed that, in some volunteers, salivary flow was affected by the thought about food [33] or by suggesting to volunteers that they drink citric acid solution instead of water [34]. In other volunteers, however, salivary flow was not affected at all [35].

This study shows significantly decreased salivation by suggestion, confirming the effect of suggestive intervention on physiological parameters shown in other studies. Hypnotic intervention has been proven to decrease heart rate, respiratory rate, blood pressure, etc. [27]. Analyzes of heart rate variability during trance showed that suggestive methods may shift the autonomous nervous system from sympathetic activity towards parasympathetic activity [36]. In conflict with our study objective, however, salivation would thus be increased. Proctor et al. found highly increased salivation by only stimulating the parasympathetic nervous system compared to only stimulating the sympathetic nervous system [37]. Emmelin showed the complicated innervations by the sympathetic nervous system. This system is controlled and regulated by different receptors that are ineffective in some salivary glands but cause low salivary flow in other glands and even high salivary flow in still other glands [38]. Salivary flow is also influenced by higher brain centers [39]. Furthermore, salivation increases and changes its composition by stimulation via mastication and gustatory stimuli [40]. Finally, as already mentioned above, thoughts about food may increase salivary flow. All these results show that salivation may be influenced by many factors.

Gemignani et al. showed increased heart and respiratory rates in anxious patients during aversive suggestion [41]. In contrast to Aubert et al., checking heart rate variability showed a shift of the sympatho-vagal indexes towards sympathetic predominance [36]. This shift could have caused the decrease in our study, but no aversive forms of suggestion were employed in our study. Many studies about changes in electroencephalography (EEG) have shown that different hypnotic suggestive interventions result in different activities in different areas of the brain reducing pain to the same extent as, for example, analgesic medications [42–44]. Even diction may influence which part of the brain becomes activated [45, 46]. Suggestive intervention is possible in many ways and may lead to many different results that can even be directly opposed.

In concordance with Wooley et al., no simple explanation exists why salivation can be decreased by hypnotic suggestion [33]. Mechanisms of controlling and regulating bodily functions are complicated, even though it is only salivary flow. Suggestive methods may influence these mechanisms. The choice of words used in suggestion seems to be important because calming words only, without any reference to the desired influence, have little or no effect at all [34]. The phenomenon that different words result in different physiological effects may be the reason for the differing results obtained by means of the direct and indirect suggestive methods in this study.

Our study only showed reduced salivation during rest periods. Mastication and gustatory stimuli were neither employed at baseline nor in suggestive measurements, i.e. we did not account for any stimuli to the oral mucosa during dental treatment. Therefore, the suggestive methods used in our trial should be applied in a dental setting to find out if these methods have the same effect during simultaneous dental treatment.

The procedure is easy and inexpensive: The equipment consists of a commercially available reproducer and earphones and the suggestion file. The volunteers in our study were neither informed about the kind of suggestion used nor about how to behave. I.e., no preceding introduction for patients should be required and a dental assistant would be able to conduct the procedure. Salivary flow should be reduced within 10 min. At this time point, we started our measurements in the study. The suggestive method furthers relaxation and does not have any secondary side effects [47]. In our study, mainly the indirect suggestive method deflected patients from the surrounding environment. Both suggestive methods can easily be reversed by de-hypnosis. No previous experience of the patients with regard to suggestion or hypnosis is required.

In combination with the rubber dam system, this method supports the reduction of moisture in the oral cavity when the highest possible level of dryness is required. Obviously, the suggestive method will not replace established techniques, such as the rubber dam system, that allows absolute dryness, protects the mucosa against corrosive liquid, aspirates root canal files in case of root canal treatment [48, 49], and protects dental personnel from infections [11]. But suggestive intervention may be helpful in supporting the rubber dam system by reducing saliva below the rubber, thus reducing the amount of suction procedures.

### Study limitations

The spitting method recommended by Navazesh et al. excludes dental diagnostics and treatment because of the forward-tilted head. The authors also tested the suction method that requires the same forward position. Contact of the suction nose with the oral mucosa is assumed to stimulate salivary flow [30]. Therefore, simultaneous measuring and dental interventions may be easily confounded. López-Jornet developed a saliva measuring procedure by wetting filter paper strips that may be an alternative for established methods but requires closure of the mouth [50].

Every volunteer used the same cup for each of his or her four recording phases including the saliva collected during the previous rounds. Evaporation decreased the amount of saliva by about 0.03 g per 5 min measuring time. The present study showed the intra-individual differences, resetting the previous value to 0. To minimize falsification, the basic value was determined a few seconds before starting a recording phase. Because the amount of saliva obtained during the preceding measurements remained in the cups, evaporation of the existing saliva from the preceding round resulted in a negative value of 0.03 g for volunteers without salivation during measuring. Therefore, these values were counted as 0 mg for statistical reasons. In all other volunteers, evaporation had the same effect on saliva in all recording phases.

Each of the 31 volunteers is a family member or friend of the author and lives in the Regensburg area. If this fact had any possible influence on the test results, it was not measured. Since all volunteers have a higher educational level than the average population, it would be interesting to know if people with a lower educational level respond better or worse to hypnosis. Furthermore, the influence of experience in hypnotic suggestion on test results has to be proved.

## Conclusion

Hypnotic suggestion represents an easy and inexpensive method for reducing salivation and is assumed to create a pleasant environment for simple and more comfortable dental treatments for both patients and dentists. Hypnotic suggestion is not intended to replace established methods for keeping off saliva from the work zone but to further decrease the activity of salivary glands. No negative side effects are known.

## Appendix

### Direct suggestion

With the help of what you hear now, you are able to put yourself in a self-hypnotic trance. Trance states are completely natural. The more you listen to what I am about to tell you, the more effectively you will experience your own self-hypnotic trance. Hypnosis is nothing more than the shift of concentration from external to inner perception.

And the more you concentrate, the closer you pay attention to what I am telling you, the more you will be able to influence the functions of your body yourself. Close your eyes now, voluntarily, because it helps you to go inside. Take long, deep, regular breaths. Deeper than you usually do. Very well. Long, deep breaths, and with your eyes closed, you can see, too. Immerge into your inner world and now focus exactly on what you see now in front of your inner eye – it does not matter whether you already perceive what you see. The only important aspect is the concentration on your inside. With your long, deep breaths, your body totally relaxes. Relax yourself all the way. Very well. Make yourself comfortable. Feel how the deeper breaths have already begun to change your body. Feel heaviness where heaviness begins, and let heaviness spread. Become increasingly heavy, very heavy, so that your whole body relaxes even more. And with those long deep breaths, you go deeper inward, and the more you transcend into the state of self-hypnosis and take a look inward, the better it will enable you to influence your body functions, because there are situations where it is easier to control the functions of the body in cooperation with the subconscious. The deeper you relax, the more you feel the heaviness, the calmer your breathing is, the easier it will be to go inside so that your subconscious is ready to accept your own suggestions. There are situations where it is better to stop the flow of saliva, because if a doctor has something to check or modify in your mouth, it is better if the mouth is very dry until the treatment is complete. Make your mouth completely dry for the duration of the treatment. Because in a dry mouth, the doctor has a better view, he is able to reach everything better, and you and your mouth are totally relaxed. Consequently, the work is done faster and cleaner, and thus the result is durable and solid. Cooperate, very relaxed and full of confidence. The more relaxed you are, the more the doctor can concentrate on his work. Entrust him with your mouth and body for the purpose of reparation from now. Continue to take long, deep breaths. Focus more and go deeper and deeper into your self-hypnosis. Go down some stairs. I will now count backwards from ten to one, and with each step, you go deeper into your self-hypnosis. Ten - nine - eight - seven - six - five - so that everything outside is and remains outside, and everything inside is more important and better - four - three - go deeper, further and further away from the here and now inside, you can control the functions of your body now - two - quiet and regular breathing, heart rate and pulse calm - one - you can shut down so far now that you can stop your flow of saliva. Stop your saliva completely now, let the salivary glands now stop production. Make your mouth dry now. Totally dry, until the work in your mouth is finished. If you make your mouth dry now, as dry as after bodily exertion, so dry that you feel thirsty, then the work in your mouth is done very quickly, easily and safely. Your whole mouth relaxes. Your tongue is comfortable, you have your mouth wide open, so that work can be done quickly and every place is easily accessible. Release your dry mouth for repair from now on; let the contacts in your mouth and on your cheek happen by being elsewhere with your attention. Abandon this situation, abandon the here and now, just leave your mouth, very dry, very comfortable, and you yourself are going into an inner idea, into an inner experience of a situation in which your saliva has vanished, and it does not have to be a march through the desert, where the sun has completely dried you out, it may also have been an encounter, where you may have met someone and have felt great need to find out further details and you did not know how far it would be possible to intensify this encounter. And you even have memories, perceptions of experiences during which your mouth was dry. Stop the flow of saliva completely now. Well done. For the duration of the treatment, you make your mouth completely dry.

### Indirect suggestion

Usually, the state of our mouth is of little interest to us, usually we do not feel anything there, usually it is not important to exercise control over the functions of the body it regulates involuntarily, and everyone has learned that when they were hungry and have seen their favorite food, even if they have only thought of it, their mouth has watered, which means that, with pure imagination, with the idea, I can unconsciously influence the normally regulated functions of my body. If the idea is suitable, if it is reasonable for me to change something, then I can affect the functions of my body through pure imagination and focus on them. And for what is to follow below, it is simpler, easier and faster if the feeling and the flow of saliva is reduced in your mouth. And it’s not so important that you know exactly how you stop the flow of saliva and what you can do to reduce the feeling in the mouth. It is much more important that you can let your thoughts wander, that you can relax, let go, think of something beautiful – feel free to perceive what you are hearing now as background noise, as if there was a radio running in the background, and you also do not need to listen to those words carefully. You also do not have to see exactly what I will describe or tell you. It is enough simply to relax, to go inside and imagine something beautiful. The more relaxed you are, the looser you feel, the more your imagination leads you out of this room and this time into a wonderful inner experience, the easier it will be for what is planned now, and the faster will we complete what is to be done in your mouth. The further you go away, the easier it will be for you to imagine: I’m somewhere else.

I would like to tell you a story: when we went on a trip to Egypt through the desert, the sun burned down completely ruthless, so that we immediately craved for shade outdoors. Especially when there was hot wind where one has their eyes squeezed and shut automatically a little bit, my mouth was dry all by itself, and we then visited this huge, massive dam. This dam supplied the entire country with electricity and dammed the water, and we could see a very small trickle at the foot of the dam, that leaked. And this huge dam had created a large lake on which a very interesting life was possible, with all the new routes, with the ships, with the road over the dam, and below the dam, you could see how the earth all was dry, parched, cracked, so that the wind kicked up clouds of dust that danced shimmering in the hot sun. Impressed by this massive human construction, we began to talk among ourselves about dams. A participant of our group reported on the work of beavers, as they cut down trees, skillfully balancing them over the water, forming a wattle with branches and can create a creek in a very short time and thereby close all the gaps in their natural dam better and better. How they stuff the gaps between the trunks and the branches, so that the beaver lake is formed, and below of their dam, the stream bed dries out, so you can walk in the stream bed and your feet remain dry. When we built dams when we were little boys, when we piled up branches, twigs and stones, full of enthusiasm in a small stream like the beavers, pasted everything nicely with clay, so that we were able to swim and splash, having fun in the pond which formed.

Yes, at that time we were much in nature, and when you’re in nature, you can experience the seasons, then you can see how the trees shed their leaves in autumn, as the sap retreats into the tree trunk, and when the fresh, cool wind comes, which then blows the leaves from the tree, sometimes even the ground at the roots of the tree is blown away, so it can be even dry in the fall. The roots are exposed and the tree itself goes into its winter sleep, as many animals do, when the time comes to retire, some form stocks, some find just a really nice place for their winter sleep, pull themselves back, curl up and turn down all body functions and dream away the coming weeks and months, until the temperature rises again and spring announces itself. And at that time, the animals dream and live a different life that is more directed inwards, they gather energy, have a rest, so that when the time of hibernation then ends, they can turn to the outer life again and increase their activities. Only when the stimulus of awakening comes from outside, they will perk up again and return to their normal everyday life in the spring.
